# Invasive Plant Species Driving the Biotic Homogenization of Plant-Frugivore Interactions in the Atlantic Forest Biodiversity Hotspot

**DOI:** 10.3390/plants12091845

**Published:** 2023-04-29

**Authors:** Wesley Dáttilo, Pedro Luna, Rafael Villegas-Patraca

**Affiliations:** 1 Red de Ecoetología, Instituto de Ecología, A.C., Xalapa 91073, Veracruz, Mexico; 2 Unidad de Servicios Profesionales Altamente Especializados, Instituto de Ecología, A.C., Xalapa 91073, Veracruz, Mexico

**Keywords:** biodiversity hotspot, biological invasions, ecological networks, frugivory networks, fruit traits

## Abstract

Although biological invasions are a common and intensively studied phenomenon, most studies often ignore the biotic interactions that invasive species play in the environment. Here, we evaluated how and why invasive plant species are interconnected within the overall frugivory network of the Brazilian Atlantic Forest, an important global biodiversity hotspot. To do this, we used the recently published Atlantic Frugivory Dataset to build a meta-network (i.e., a general network made of several local networks) that included interactions between 703 native and invasive plant species and 331 frugivore species. Using tools derived from complex network theory and a bootstrap simulation approach, we found that the general structure of the Atlantic Forest frugivory network (i.e., nestedness and modularity) is robust against the entry of invasive plant species. However, we observed that invasive plant species are highly integrated within the frugivory networks, since both native and invasive plant species play similar structural roles (i.e., plant status is not strong enough to explain the interactive roles of plant species). Moreover, we found that plants with smaller fruits and with greater lipid content play a greater interactive role, regardless of their native or invasive status. Our findings highlight the biotic homogenization involving plant–frugivore interactions in the Atlantic Forest and that the impacts and consequences of invasive plant species on native fauna can be anticipated based on the characteristics of their fruits.

## 1. Introduction

Biological invasions are one of the most important threats to biodiversity on Earth and generate great economic losses for society [1]. In recent decades, biological invasions have significantly increased all around the world because of globalization and climate change [2,3]. Although biological invasions are rapidly escalating worldwide, most of the accumulated knowledge of invasive species is focused on their colonization and impact on native biodiversity and management, but often ignores the biotic interactions they play in the environment [4,5,6]. However, all species are linked through the web of life and their interactions represent one of the main components of biodiversity because they regulate functions, from populations to entire ecosystems [7,8]. Thus, studying species in isolation without their biotic interactions can mask information and is not sufficient to predict an integrative response to biological invasions.

Some of the most remarkable biotic interactions in tropical regions are frugivory and related to seed dispersal by animals [9,10], mainly because estimates suggest that up to 90% of tropical plant species produce fleshy fruits that are used by frugivorous animals [11]. Moreover, frugivory activity and seed dispersal are crucial for plant recruitment and growth in tropical forests, as they affect the spatial recruitment patterns of plant communities [12,13,14]. Interactions between frugivores and plants also influence how species’ traits evolve; for example, seed size in a forest fragment is related to the body size of the frugivores living there [15,16]. Certainly, frugivory activity is directly related to the ecological and evolutionary dynamics of ecosystems [17,18], and understanding which factors are disrupting these types of interactions is a challenge and an opportunity to understand how biological communities are vulnerable to biological invasions [19,20,21]. For example, understanding the way invasive plant species couple within well-established native frugivory interactions has the potential to help us understand the establishment and spread of non-native species in new regions [22,23].

An interesting framework for studying the way invasive and native species interact in species-rich environments uses tools derived from complex network theory [24]. In this network of species interactions, species are represented as nodes and their interactions by links that connect interactions between native and invasive species [8]. In recent decades, the use of interaction networks has helped us understand the structure and organization of interactions between plants and frugivores and their vulnerability to different types of environmental disturbances [25,26]. For example, there is evidence that highly invasive fruit-bearing plants can change the structure of seed dispersal networks between birds and plants [27], which can lead to non-native plants competing with native for dispersal services [28]. However, like native species, plant invasive species also differ in their spatial abundance, fruit size and nutritional traits, which ultimately affect selection and consumption by frugivorous animals [29,30]. In fact, fruit traits and density are important factors that shape structural organization of frugivory networks [31]. Therefore, understanding how invasive plant species are connected within native frugivory networks and how the characteristics of their fruits affect their importance within such networks can be crucial to understanding biological invasions and to predicting how and why species will invade certain regions [32,33].

As species differ in their ability to disperse, colonize and establish themselves in new environments, there are some environments that are more vulnerable than others to biological invasions (e.g., islands and biodiversity hotspots) [34]. In this sense, the Brazilian Atlantic rainforest is a global biodiversity hotspot with an exceptional concentration of endemic species and a restricted geographic distribution, and is highly threatened by human activities [35,36]. In fact, ~70% of Brazil’s population is concentrated in the Atlantic Forest region, where the country’s largest cities are located [37]. Because human activity is one of the main factors responsible for enhancing biological invasions, the high human population density within the Atlantic Forest domain, together with different factors associated with other anthropogenic, historical, and natural factors, have generated a high risk from the spread and establishment of non-native species in the Atlantic Forest [38,39,40]. Although the introduction of non-native species in the Atlantic Forest has occurred since the 1500s with the arrival of European colonization, the number and record of invasive species of different taxonomic groups (i.e., plants, fish, birds, mammals, reptiles and insects) has grown exponentially over the last 70 years which has led to a great homogenization of biota (i.e., the increase in spatial community similarity over time) [40]. Therefore, we urgently need to understand how invasive species are integrating with other native species in this important global biodiversity hotspot.

In this work, we evaluated how invasive plant species are interconnected within the native frugivory network of the Brazilian Atlantic Forest. Specifically, we addressed the following issues: (i) What structure emerges from frugivory networks when considering only native plant species, invasive plant species, and the meta-network (i.e., a general network build using both native and invasive plant species)?; (ii) Do native and invasive species contribute equally to the overall meta-network structure?; (iii) Does the interactive role of native and invasive species differ within the frugivory meta-network?; (iv) Do the size and concentration of lipids in fruits determine the importance of plant species within the meta-network? and; (iv) What is the magnitude of variation of frugivore composition interacting with native and invasive plant species? Overall, our findings advance the knowledge of how invasive plant species are interconnected with native species in a large tropical biome. We hope that our contribution will stimulate new studies on species interaction networks and biological invasions over broad spatial scales, therefore evaluating to what extent our findings can be generalized to other types of plant–animal interactions and biodiversity hotspots.

## 2. Methods

### 2.1. Data Set

**Figure 1:**
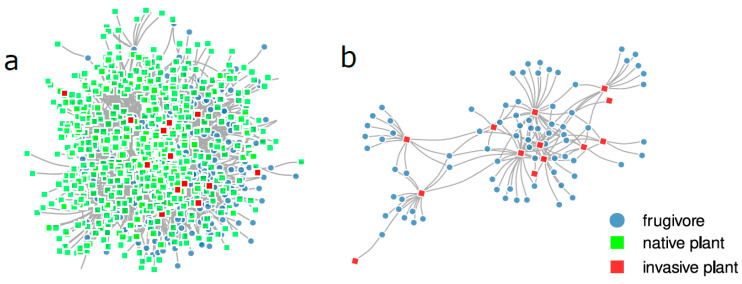
(**a**) Meta-network which includes all interactions between native and invasive plants and frugivores in the Atlantic Forest biome; (**b**) Plant–frugivore subnetwork, including only the interactions between involving invasive plant species and frugivores. Each node denotes a species and the links denote interactions between them. Circles denote frugivore species while red squares denote invasive plant species and green squares represent native plant species. These networks were drawn by using the data obtained from the Atlantic Frugivory Dataset [41] after filtering the interactions by the status of plant species (i.e., native and invasive).

We used the Atlantic Frugivory Dataset [41] which comprises 8320 frugivory interactions between 331 vertebrate species and 788 plant species. Here we used this most comprehensive data set available for a tropical ecosystem to build a meta-network in which we only included interactions between frugivores and native and invasive plants. After filtering the interactions by the status of plant species (i.e., native and invasive plants), our final dataset included 703 plant species (690 native and 13 invasive) and 331 frugivores (232 birds, 90 mammals (mainly bats), 5 fish, 1 amphibian and 3 reptiles). The interactions included in this study are located between Latitudes −1.4527° and −47.109 within the Atlantic Forest biome, which is a biodiversity hotspot highly threatened by anthropogenic pressures [42,43] (Figure 1).

To test if the interactions between frugivores and invasive plant species have an impact on overall interaction dynamics we first built a meta-network *A*, where a_ij_ = number of interactions between plant species *j* and frugivore species *i*. Then we obtained two subnetworks from the main meta-web, one including only interactions between native plant species and all frugivores (native interaction network) and another including only interactions between invasive plants species and all frugivores (invasive interactions network) (three networks in total).

### 2.2. Data Analysis

To test if the interactions established between frugivores and invasive plant species have an impact on the overall frugivore meta-network of the Atlantic Forest, we first tested if the meta-network and the native and invasive subnetworks showed a non-random structure. For this we measured its nestedness and modularity. Nestedness is a descriptor that indicates that the interactions within a network have a hierarchical arrangement, in which species with fewer interactions often interact with a proper subset of the partners of more connected species. Modularity describes if within each network there are groups of species interacting more strongly with each other than with species in other groups in the network. To measure nestedness, we used the *NODF* index (nestedness based on overlap and decreasing fill) [44] and for modularity we used the QuanBiMo algorithm [45]. The NODF index ranges from 0 (non-nested) to 100 (perfectly nested). The *Q* index uses an algorithm that calculates the modularity for weighted networks using the Likelihood and Simulated Annealing-Monte Carlo approach, which ranges from 0 (no subgroups) to 1 (completely separated subgroups) [45]. To test the significance and to standardize the differences in connectance and heterogeneity of interactions between networks, we calculated the Z-scores of the values of nestedness and modularity of the three networks (Meta-network, native species subnetwork, and invasive species subnetwork). The Z-transformed score is defined as follows: Z = [x − µ]/σ, where x is the observed index value, µ is the mean of the values from simulated matrices, and σ is the standard deviation of the values from simulated matrices [44]. The simulated matrices used to calculate µ and σ were generated by different null models depending on the index (NODF or QuanBiMo). For nestedness, we generated 1000 networks according to Null Model II, in which the probability of an interaction occurring is relative to the observed number of interactions of both plant and frugivore species. For modularity, we generated 1000 networks according to the null model Patefield [46], which holds the marginal totals constant (i.e., observed row and column totals) while allowing network connectance to vary.

After measuring overall network structure, we tested if native and invasive species contribute equally to overall meta-network structure (nestedness and modularity) and if the interactive role of each species (native and invasive) changes within the meta-network. For this analysis, we estimated the degree to which the interactions of plants species (either native or invasive) increase or decrease the network overall nestedness compared to random expectations [47]. In addition, we recorded the network roles of species in the modular structure by calculating (i) the standardized within-module degree (*z_i_*), which is a measure of the extent to which each species is connected to other species in its module, and (ii) the among-module connectivity (c_j_), which measures how evenly distributed the interactions of a given species are across modules [48]. The interactive role of plant species was measured by considering different network centrality descriptors: betweenness, closeness, species strength, species degree (*k*), and Katz centrality. Betweenness describes the role of a species as a potential bridge to connect other species by measuring the shortest paths that passes through the target species [49]. Closeness measures the average length of the shortest path between a target species and all other species in the networks. Species strength is the sum of dependencies of each species and aims to quantify a species’ relevance across all its partners. Species degree is the number of interactions each species establishes with other species in the network. Katz centrality measures the relative degree of influence of a species within a network by measuring the number of immediate neighbors and the direct and indirect paths to other species in the network (plants or frugivores) [50].

The role of a species within a complex and diverse network may depend on various factors. To estimate its importance, multiple centrality measures can be considered to robustly assess a species’ role within large networks. In this study, we utilized principal component analysis (PCA) to summarize and combine several centrality indexes, including betweenness, closeness, species strength, species degree and Katz centrality, into a single value [51]. The first principal component score (PC1) accounted for 66.9% of the variability, indicating complementarity among the five-centrality metrics and reinforces the use of PC1 as a measure of centrality. Therefore, we used PC1 to reduce the five-dimensional space to a single generalized centrality index. It is important to note that scores on PC1 were positively correlated with the selected centrality measures. Hence, species with high PC1 scores are indicative of a highly interactive role within the network, being connected to other species through multiple direct and indirect pathways, while low PC1 scores suggest species with a low interactive role (i.e., peripheral species).

We then compared the structural and interactive roles of the native plants and invasive plants of the meta-network using the values obtained by measuring their contributions to nestedness, species roles in modules (*z_i_* and *c_i_*), and the interactive role of species (PC1). Because the number of native plants in the meta-web is greater than the number of invasive species (690 native species vs. 13 invasive species), any direct comparison between the values of both groups would be unbalanced and biased due to the differences in species number. Therefore, instead of using a conventional statistical analysis we employed a Jackknife procedure. For this, we randomly selected small subsets of the values of native species (N = 13) and subsequently tested if there were statistical differences between the 13 values of invasive species and the randomly selected values of native species. The comparisons were carried out with a Student’s *t*-test and the random values were selected 500 times (i.e., one *t*-test for each iteration). Then we estimated the *p*-value based on the number of times that we found statistical differences between the values of each network index (contribution to nestedness, species’ roles in modules, and the interactive nature of roles within species) of the interactions of native and invasive plant species (frequencies of statistical significative models/500). With this, we ensured that our comparisons were balanced and therefore we avoided any bias in comparing 690 species with only 13.

Moreover, to examine which other factors could determine the role of a species within the studied plant-frugivore meta-network, we compared the sizes of fruits of native and invasive species using the same Jackknife procedure used for the comparisons of the contribution to network structure and the interactive roles of species. We also assessed whether there was a relationship between the interactive role of a plant species (PC1) with the size of its fruit (diameter and length), its lipid content (low, mid, or high content), and the status of the plant (native or invasive). For this, we fitted a generalized linear model with a Gamma error distribution (inverse link function), where the interactive role of a species (PC1) was the dependent variable and fruit size, lipid content, and status were independent variables. Fruit size and lipid content values were obtained from the Atlantic Frugivory dataset [51]. In this case, the lipid content was a categorical factor for ranking the fruits based on their lipid concentration in dry weight: fruits with low lipid concentration have <10% of lipid, fruits with medium concentration between 10 to 20% of lipid, and fruits with high concentration have >20% of lipid [51].

Finally, to assess the variation in the composition of frugivore species interacting with native and invasive plants species, we calculated the beta diversity of frugivores between both groups of plants. We used the beta diversity framework proposed by Baselga [52,53], in which we partitioned *β_jac_* in two components, *β_sp_* turnover (species change) and *β_ne_* (species gains/loss), using the Jaccard dissimilarity index.

For all analyses we used R [54], version 4.2.2 with the packages bipartite [55], vegan [56], igraph [57], and betapart [52].

## 3. Results

We found 13 invasive plant species in the Atlantic Frugivory Dataset (i.e., *Acacia auriculiformis*, *Archontophoenix cunninghamiana*, *Artocarpus heterophyllus*, *Hovenia dulcis*, *Ligustrum japonicum*, *Ligustrum lucidum*, *Litchi chinensis*, *Livistona chinensis*, *Morus alba*, *Morus nigra*, *Musa dasycarpa*, and *Musa rosacea*), which represented 7 families (i.e., Arecaceae, Fabaceae, Moraceae, Musaceae, Oleaceae, Rhamnaceae, and Sapindaceae) and 146 unique interactions. Our plant-frugivore meta-network exhibited a nested (NODF = 10.40, *p* < 0.0001, Z-score = 385.53) and modular structure (Q = 0.4321, *p* < 0.05). The subnetwork containing only interactions between native plants and frugivores exhibited a nested (NODF = 10.45, *p* < 0.001, Z-score = 376.89) and modular structure (Q = 0.4279, *p* < 0.05). The subnetwork containing only the interactions between invasive plants and frugivores exhibited a nested structure (NODF = 23.15, *p* < 0.0001, Z-score = 12.08), but it showed no modular structure (i.e., no subgroups of species were detected, Q = 0.4833, *p* > 0.05).

**Figure 2:**
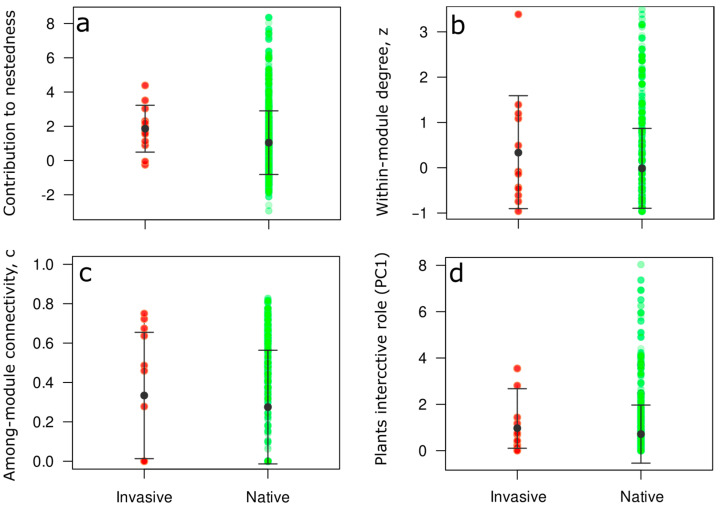
Comparisons between the values of plants’ (**a**) contributions to nestedness, (**b**) within-module degree, (**c**) among-module connectivity, and (**d**) plants’ interactive roles. The black point denotes the mean for invasive plants and native plants (mean of 500 subsamples), and the lines denote the standard deviation. Each red point denotes an invasive plant species and green points denote the subsampled native plant species.

When we assessed the differences in the contributions to nestedness, species roles in modules (z_i_ & c_i_) and the interaction of role species (PC1) between native and invasive species, we observed that native (mean of 500 subsamples ± SD of 500 subsamples = 1.03 ± 1.8) and invasive (mean ± SD = 1.85 ± 1.36) plant species made similar contributions to network nestedness (*p* = 0.8; Figure 2a). Moreover, in the case of the contribution to modularity, we observed that native and invasive species had similar within-module connectivity (*z_i_*) (native = 0.01 ± 0.91; invasive = 0.34 ± 1.24, *p* = 0.99; Figure 2b) and similar among-module connectivity (*c_i_*) (native = 0.28 ± 0.27; invasive: 0.33 ± 0.32, *p* = 0.99; Figure 2c). For the interactive roles of plant species, we observed that both native (0.75 ± 1.33) and invasive species (1.38 ± 1.28) had similar roles within the meta-network (*p* = 0.78; Figure 2d).

**Figure 3:**
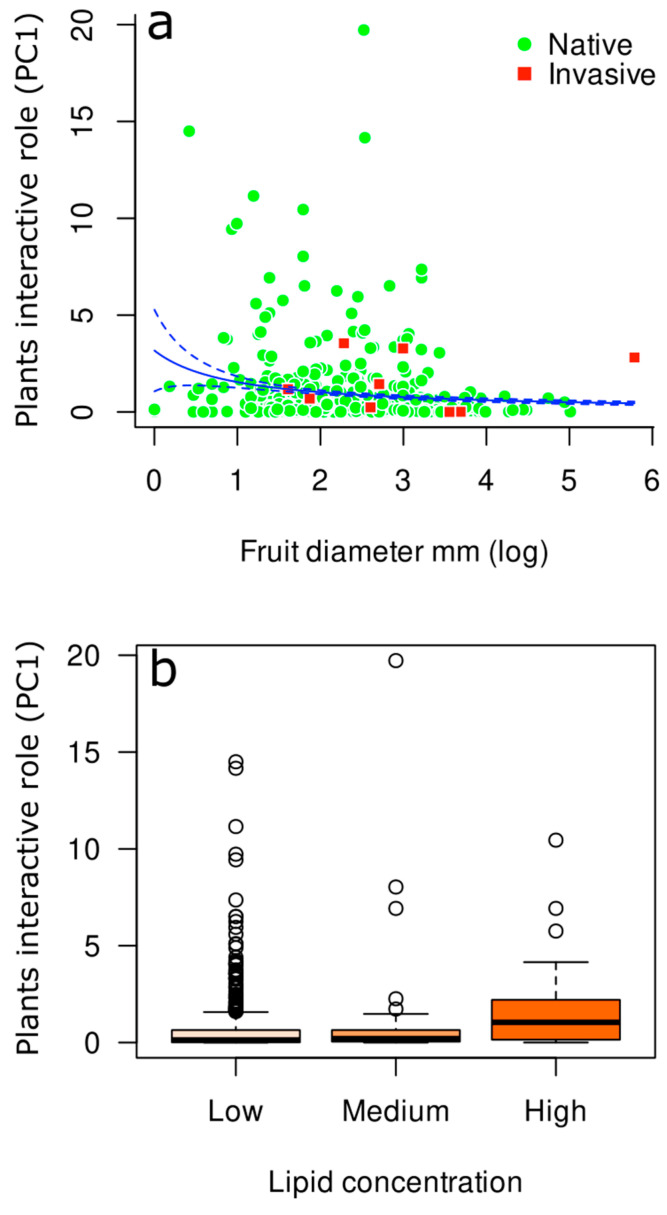
(**a**) Relationship between plants’ interactive roles (PC1) and fruit diameter mm (log). (**b**) Comparison between the values of plants’ interactive roles (PC1) across the different categories of lipid content. The values for fruit size and lipid content are available in the Atlantic Frugivory dataset [41].

When we evaluated whether other factors beyond the status of the plants explained the interactive roles of plants within our meta-network, we found that the diameter of the fruit and its lipid content explained its interactive role in the network. The diameter of native species fruits (mean of 500 subsamples ± SD of 500 subsamples = 5.35 ± 4.32 mm) was similar to the diameter of invasive species fruits (mean ± SD = 8.07 ± 6.36 mm, *p* = 0.49). However, we observed that plants with smaller fruits (diameter in millimeters) are consumed more by frugivores (i.e., more central position within the network) (χ^2^ = 36.66, df = 437, *p* = 0.002) (Figure 3a). We also observed that fruits with greater lipid content are consumed more by frugivores (χ^2^ = 20.26, df = 435, *p* = 0.02) (Figure 3b). The status of the plant species (native or invasive) was not relevant to determining whether it was consumed more or less by the frugivores included in this study (χ^2^ = 4.27, df = 435, *p* = 0.3). Finally, we observed that the variation in the composition of frugivores between native and invasive plants was high (*β_jac_ =* 0.78) and was mainly explained by changes in the number of species that interact with native and invasive species (*β_ne_ =* 0.66), instead of by changes in the composition of species (*β_sp_* = 0.12).

## 4. Discussion

Here we showed how invasive and native plant species similarly contribute to the organization of a diverse plant–frugivore network in the Atlantic Rainforest Biome. Furthermore, we demonstrated that traits associated with fruit size and nutrient concentration are factors that explain the importance of plant species within plant–frugivore interaction networks, regardless of whether the plant is native or invasive. Our results show evidence of how invasive plants are already integrated in the frugivory dynamics of the Atlantic Forest, since our dataset was extracted from 166 published and unpublished sources spanning 1961–2016. These findings help us better predict the risk and consequences of future invasions and the persistence of native biodiversity in the Atlantic Forest biodiversity hotspot.

In recent decades, understanding of the structure of interaction networks between plants and frugivores has grown and attracted the attention of ecologists around the world [58,59,60,61]. Here, we showed that both the meta-network (involving both native and invasive species) and the network considering only native species exhibit nested and modular patterns of species interactions. These results are similar to many studies that show that plant–frugivore networks exhibit these types of non-random organization [62,63,64]. However, when we only considered the interactions involving the invasive species, we observed that the network was nested but not modular. Nowadays, we know that one of the main factors that determines the nested pattern in species interaction networks is the difference in relative species abundance, because abundant species should interact most frequently, while less abundant species tend to interact with abundant species but will rarely interact among themselves [65,66,67,68]. Thus, abundance-based processes could sufficiently explain the nested pattern in networks involving only invasive plant species. The lack of a modular pattern in the network involving only invasive plants is possibly because this is a very small network (compared with the whole meta-network) and the few species do not interact according to different dispersal syndromes (i.e., phenotypic traits correlated with dispersal). The interactions of invasive species have also been established in more recent times and do not represent long evolutionary and coevolutionary histories that could generate a high specialization, compartmentalization and, consequently, a modular pattern [62], as we observed for the meta-network and the network involving only native plant species. Thus, our results indicate that the pattern of organization in the Atlantic Forest frugivory network is robust against the entry of invasive plant species and the modularity of the meta-network might be a pattern emerging from highly diverse networks.

Our results also indicate that both native and invasive species play similar roles (i.e., contribute to nestedness and modularity) within the frugivory meta-network of the Atlantic Forest, and that plant status is not strong enough to explain the importance of species (i.e., interactive role) within the network. Similarly, in a study performed by Heleno et al. [69] in the Azores, the authors showed that exotic species were as important as native species for dispersers, indicating that birds depended equally on native and exotic fruits, regardless of their abundance in their study area. Contrary to our results, evidence has shown that invasive plant species can alter the structure of seed dispersal networks [27], with potential implications for ecological and evolutionary dynamics [70]. This calls our attention to the fact that biological invasions can have negative consequences for native species and communities and that introduced plant–frugivore interactions have increased sevenfold over the past 75 years around the world [71]. It is very important to highlight that in this study we only evaluated the roles of native and invasive plant species within the frugivory meta-network and found that both species exhibited similar roles. However, this does not mean that any effects could not occur in the future or that they do not exist through other negative aspects of invasive species on native interactions (e.g., seed dispersal effectiveness), leaving this topic to future investigations. We must also consider that the frugivory network of the Atlantic Forest is highly diverse, which would act as a buffer preventing new species from occupying important roles within the network that are already well coupled and developed by native species over time.

Here, we showed that plants with smaller fruits and with greater lipid content play greater interactive roles within frugivory meta-networks, regardless of their native or invasive status. This is because plant status (native or invasive) was not a significant variable within our generalized linear model. In fact, different studies have shown that the amount of lipids in fruits is an important factor that structures the ways plants and frugivores interact in nature [72,73,74], mainly because this characteristic contributes to make them highly energetic and attractive for different frugivore species [75]. These findings are in line with the optimal foraging theory, since it is expected that frugivores should select high-caloric lipid-rich fruits to offset the energetic costs of foraging (i.e., increase benefit and minimize cost) [76,77]. In addition to the amount of lipids, we found that plant species with small fruits can attract a great variety of frugivore species, which gives these plant species highly interactive roles within the frugivory meta-network, as shown in this study. Previous studies also showed that plant species with smaller seeds tend to be more important to network organization [63,72]. This is possibly because small fruits can be eaten by many frugivores, for example, birds with large or small beaks, whereas large fruits would only be eaten by frugivores with beak or mouth gapes wide enough to swallow the seed or the fruit [11]. This fruit size threshold in frugivory interaction patterns can lead to different ecological (e.g., dispersal quality) and evolutionary (e.g., fruit size selection) consequences [16,78]. Therefore, both invasive and native plant species seem to be equally used resources by Atlantic Forest frugivores, as long as they have small fruits and high concentrations of lipids. In this sense, we also found no evidence that frugivore species interact more strongly with plants of a given status since native and invasive plant species establish interactions with similar frugivore species, but native plants interact with a larger group of frugivores than invasive species. These results are in accordance with the ‘‘Fraction Similarity Hypothesis’’, which predicts that the success of invasive species in an environment benefit from existing native mutualistic interactions [33]. In other words, fruits of invasive plant species should exhibit the same characteristics as native species and, therefore, would be functionally equivalent to fruit-eating birds. Overall, our results highlight that the impacts and consequences of invasive plant species on native fauna can be anticipated based on the characteristics of their fruits.

In this study, we showed how and why invasive plant species are connected in native plant–frugivore interactions in the Atlantic Rainforest biome. In general, we found that invasive and native species contribute equally to the organization of frugivory networks and that fruit size and lipid content are the most important factors to determine the interactive roles of plants within the meta-network, regardless of whether the plant species is native or invasive. We also observed that the frugivore species that interact with the invasive plant species form a subset of the frugivore species that interact with the native plant species. In short, our findings indicate a biotic homogenization in the interactions between plants and frugivores in the Atlantic Forest, mainly due to high similarity in the importance of invasive and native plants within the frugivory networks, in addition to the overlapped taxonomic composition of the frugivores in which they interact. However, specific ecological and evolutionary consequences of this biotic homogenization of species interactions at different spatial and temporal scales of the Atlantic Forest remain unexplored and future work should address this topic, including the effects of frugivorous species on plants.

## Data Availability

The data presented in this study are openly available in Data Papers sections of Ecology at https://doi.org/10.1002/ecy.1818, accessed on 8 March 2023, reference number ecy.1818.
